# Ambivalence: A Key to Clinical Trial Participation?

**DOI:** 10.3389/fonc.2018.00300

**Published:** 2018-08-10

**Authors:** Janice A. Chilton, Monica L. Rasmus, Jay Lytton, Charles D. Kaplan, Lovell A. Jones, Thelma C. Hurd

**Affiliations:** ^1^Pharmacy Administration and Administrative Health Sciences, Texas Southern University, Houston, TX, United States; ^2^Adult Mental Health and Wellnes, University of Southern California, Los Angeles, CA, United States; ^3^Haimovitch Center for Science in the Human Services, University of Southern California, Los Angeles, CA, United States; ^4^Professor Emeritus, University of Texas MD Anderson Cancer Center, Houston, TX, United States; ^5^Department of Surgery, University of Texas Health Science Center at San Antonio, San Antonio, TX, United States

**Keywords:** ambivalence, clinical trials, disparities, motivational interviewing, trust

## Abstract

Trust exerts a multidimensional influence at the interpersonal level in the clinical trials setting. Trust and distrust are dynamic states that are impacted, either positively or negatively, with each participant-clinical trials team interaction. Currently, accepted models of trust posit that trust and distrust coexist and their effects on engagement and retention in clinical trials are mediated by ambivalence. While understanding of trust has been informed by a robust body of work, the role of distrust and ambivalence in the trust building process are less well understood. Furthermore, the role of ambivalence and its relationship to trust and distrust in the clinical trials and oncology settings are not known. Ambivalence is a normal and uncomfortable state in the complex decision making process that characterizes the recruitment and active treatment phases of the clinical trials experience. The current review was conducted to understand the constructs of ambivalence as a mediator of trust and distrust among vulnerable, minority participants through different stages of the oncology clinical trials continuum, its triggers and the contextual factors that might influence it in the setting of minority participation in oncology clinical trials. In addition, the researchers have sought to link theory to clinical intervention by investigating the feasibility and role of Motivational Interviewing in different stages of the clinical trials continuum. Findings suggest that ambivalence can be processed and managed to enable a participant to generate a response to their ambivalence. Thus, recognizing and managing triggers of ambivalence, which include, contradictory goals, role conflicts, membership dualities, and supporting participants through the process of reducing ambivalence is critical to successfully managing trust. Contextual factors related to the totality of one's previous health-care experience, specifically among the marginalized or vulnerable, can contribute to interpersonal ambivalence. In addition, changes in information gathering as a moderator of interpersonal ambivalence may have enormous implications for gathering, assessing, and accepting health information. Finally, motivational Interviewing has widespread applications in healthcare settings, which includes enabling participants to navigate ambivalence in shared-decision making with their clinician, as well as executing changes in participant behavior. Ultimately, the Integrated Model of Trust can incorporate the role of therapeutic techniques like Motivational Interviewing in different stages of the clinical trials continuum. Ambivalence is a key component of clinical trial participation; like trust, ambivalence can be managed and plays a major role in the management of trust in interpersonal relationships over time. The management of ambivalence may play a major role in increasing clinical trial participation particularly among the marginalized or the vulnerable, who may be more susceptible to feelings of ambivalence.

## Trust, distrust and ambivalence

The decision to trust or distrust, in the context of clinical trials and oncology care, is the product of the interactions between trust, distrust, and ambivalence. Hillen et al. and Hudson et al. (1, 2) Many factors influence this decision such as prior participant trust experiences both within and outside of the health-care system, provider factors that influence trust such as communication, honesty, competence, and confidence, health system, and institutional factors. Most important is the relationship between the participant and clinical trials team members that is constantly evolving as a result of ongoing treatment, system related factors, and interparticipantal engagement (3–9).

The relationship between trust, distrust, and ambivalence has been evolving in recent years. Lewicki et al. (10) first proposed that trust and distrust coexist and are moderated by ambivalence. The model was expanded to include affect based trust as well as attributes, characteristics and contextual factors associated with distrust (11–13). Reich et al. (14) have proposed that ambivalence is embedded in the definition of trust in which one accepts vulnerability (negative attribute) in exchange for “positive expectations of the intentions or behavior of another in the future” (positive attribute). Trust results as the consequence of accepting ambivalence rather than vulnerability. Therefore, its relationship with trust and distrust may be more intimate than previously accepted. More recently, the Integrated Model of Trust has incorporated and adapted the Lewicki model to the clinical trials setting (15). Trust and distrust are dynamic states that are impacted, either positively or negatively, with each participant-provider interaction in the clinical trials setting. The psychological contract serves as the vehicle through which trust and distrust are renegotiated. Extrapolating this to the clinical trials setting, Hurd et al. suggest that ambivalence, like trust, must also be managed. It is subject to the same tensions that exist between participants and clinical trials team members during the recruitment, retention, surveillance phases of the clinical trials experience as well as the care transition points that occur during transition from the referral to treatment and treatment to surveillance phases (15, 16).

Although knowledge of minority participant trust and distrust in clinical trials and oncology care is expanding, there are significant gaps in our knowledge of the role of ambivalence in these settings. This review will explore the construct of ambivalence, examine the triggers of ambivalence at the individual level and consider the impact of contemporary and societal factors on ambivalence that can affect participation rates in clinical trial studies will also be explored. Finally, this review will consider how ambivalence can be mitigated through evidence-based interventions, such as Motivational Interviewing, and its potential applications in clinical trial settings. Specifically, Motivational Interviewing has widespread applications in healthcare settings, including assisting patients to ameliorate ambivalence around shared-decision making with their clinician, as well as changes in participant behavior (17). Ultimately, the Integrated Model of Trust can incorporate the role of therapeutic techniques like Motivational Interviewing in different stages of the clinical trials continuum.

## Ambivalence

The presence of simultaneous, strong positive or negative attitudes toward a participant, object or situation (entity) defines ambivalence (10). These attitudes are based on differing qualities or characteristics that are inherent in, or ascribed to, something or someone (18). Ambivalence arises when an individual's expectations of a participant, object, or situation are opposed to what is encountered or displayed. It creates a psychological tension or conflict (subjective ambivalence) that occurs when the participant is evaluating their attitudes toward an entity as well as tension arising from the response to the attitude associated with an entity (structural ambivalence) (19).

Ambivalence is often considered synonymous with uncertainty, ambiguity or neutral attitudes. However, it is distinct from these due to the presence of *strong* attitudes. Specifically, uncertainty is associated with positive or negative attitudes generated with diminished confidence. Ambiguity arises from a lack of familiar cues, cues that have both positive and negative characteristics or an abundance of cues that must be evaluated and categorized. Neutral attitudes are characterized by the absence of both positive and negative attitudes (20).

Individuals receive both positive and negative information during interactions with others which must be evaluated and ultimately assigned a positive or negative attribute. Positive information results in assigning positive attributes to the participant, object, or situation while negative information results in assigning negative attributes. The quality and volume of positive or negative attributes determines the strength of the positive or negative attitude toward the participant, object or situation (21, 22). Ambivalence arises when a person's evaluations of an object, person or situation, and the resulting attributes that are repeatedly experienced over time, are inconsistent. The Threshold Model of Ambivalence posits that ambivalence does not exist in either a present or absent state (23). Rather, it manifests over time once a critical threshold of conflicting reactions is reached resulting in a participant feeling strong positive and negative attitudes, i.e., familiarity breeds ambivalence. The threshold varies with the context and relevance of the object, person or situation to the person experiencing ambivalence. In this model the rate at which ambivalence increases is slowed as the amount of conflicting information increases once the threshold is crossed (23).

Ambivalence exerts both positive and negative effects which can result in both good and bad outcomes (24). Historically it was thought to be a negative state associated with high uncertainty, negative thinking, bias, narrow thinking, reduced ability to decide, resistance to change, decreased confidence in attitudes, diminished processing of relevant information, decisional paralysis and behavioral inflexibility (14, 25). However, recent work has shown that ambivalence can have positive effects. It increases information processing and can increase cognitive breadth by forcing people to consider multiple perspectives and unlearn inaccurate information/perceptions. Furthermore, it increases behavioral flexibility and adaptability to enable change in a participant's viewpoint or actions resulting in increased resilience to a stressor event and lower ambivalence (26–28). Perhaps, most importantly, the positive effects of ambivalence cause people to leverage their collective wisdom by embracing the known and unknown, acknowledging that the outcome of a situation is unpredictable and linking prior knowledge and experiences to resolution of ambivalence (29).

Ambivalence creates a psychologically stressful state of varying intensity and the intensity of opposing attitudes is directly linked to the ascribed importance of the issues underlying ambivalence to the participant (30). Individual susceptibility to ambivalence varies and the response to it may be conscious or unconscious. Certain individual characteristics like knowledge based investigative thinking, fear of invalidity, low need for knowledge, age, and thoughtful approach to self-emotion are associated with ambivalence (14).

The overarching goal of a participant's response to ambivalence is to decrease the psychological discomfort to a manageable level by resolving the incongruities between the positive and negative information. People use three strategies to decrease ambivalence- heuristic and systematic processing, selectivity and compensatory perceptions of order. Heuristic processing applies simple rules to judge information which enables them to process conflicting and non-conflicting information with the least expenditure of cognitive effort (Least Effort Principle) and high confidence in their final judgement (Sufficiency Principle) (31). A participant's confidence in their final judgment is constantly self monitored and depends upon the participant and their motivational state. Heuristic processing is not sufficient if a high level of confidence in a final judgement is needed. If ambivalence cannot be reduced to a subthreshold level systemic processing is employed. This approach requires comprehensive analysis and scrutiny of any information that will reduce or resolve ambivalence and is both cognitively and temporally consuming. The outcomes of each of these approaches are distinct. In summary, the heuristic approach is efficient but does not produce durable perceptions and attitudes that are stable over time. Conversely, systemic processing produces strong stable attitudes and perceptions and is a better predictor of future behaviors (25).

Selectivity, occurs when a person only selects and retains the information that supports their underlying positive or negative attitude. Prior positive or negative experiences do not influence the selectivity process, because it is employed when a participant lacks knowledge about an issue (32). Selectivity might be operative during the recruitment phase of the clinical trials continuum. Thus, a participant with knowledge of prior research abuses and who experiences both positive and negative interactions during trial recruitment might selectively focus on negative rather than positive attributes of the experience.

A third approach used to cope with ambivalence is compensatory perceptions of order. Compensatory perceptions allow people to avoid changing their underlying positive or negative attitudes about an entity (33). Ambivalence is a disordered and incongruous state and order is necessary to effectively address it. To accomplish this, a person ascribes false characteristics and/or qualities to the positive or negative information based upon incorrect perceptions, or ascribes to conspiracy theories that support negative perception. Finally, it can also occur after having an experience that confirms an underlying positive or negative belief.

Processing and managing ambivalence enables a participant to generate a response to their ambivalence. Ashforth et al. (34) have proposed four distinct responses to ambivalence that people employ based upon the situation in which ambivalence arises: avoidance, compromise, domination and holism (Table 1). Each response occurs along a continuum of positive and negative attitudes and, in turn, can have either positive or negative outcomes.

**Table 1:** Responses to ambivalence (34).

	**Positive orientation toward clinical trials participation**		
**Negative orientation toward clinical trials participation**	Low	Intermediate	High
Low	Avoidance		Domination
Intermediate		Compromise	
High	Domination		Holism

*Adaptive responses to ambivalence among minority participants in clinical trials*.

Avoidance is used when a participant believes that the source of ambivalence must be endured instead of managed, is situationally influenced and does not involve overt behaviors (35, 36). It allows participants to rely on defense and coping mechanisms to reduce the psychological tensions directly associated with the source of ambivalence. The defense mechanisms (denial and splitting) are unconscious and protective in nature while coping mechanisms are solution focused, conscious and intentional. Denial allows participants to reject, reinterpret, forget or diminish disagreeable information. Splitting enables them to separate the positive and negative orientations, suppress one or the other and reduce ambivalence. Avoidance does not address the root cause(s) of ambivalence and can obscure them from the participant. However, it can be useful when the participant cannot address the underlying issue effectively (power imbalance) or when the positive and negative feelings are not in conflict as might occur in subthreshold phases of the threshold model (23).

Domination, also a defense/coping mechanism, occurs in the hybrid settings of high negative and low positive and low negative and high positive evaluation. It is a reactive mechanism which unlike avoidance is associated with taking action. When utilized the participant exaggerates either the positive or negative evaluation so that it dominates the other orientation and decreases ambivalence. The dominated element continues to exist and may become dominant over time as situations change. Domination is useful when a participant must choose between two mutually exclusive actions, one orientation is counterproductive or the core elements of a positive or negative evaluation don't need to be maintained. However, domination can create highly dysfunctional states when both positive and negative orientations must be maintained or the initial orientation is salient or the decision is based solely on relieving the psychological discomfort (34).

Compromise occurs when positive and negative evaluations are both accommodated through reciprocal concessions. It is action oriented and can be accomplished by evaluating the positive and negative attributes and finding a middle ground that is neither positive nor negative or keeping compatible elements of both the positive and negative attributes. Compromise is most effective when neither the positive nor negative attributes are sufficient to favor one attitude over another but the core attitudes can be maintained and when the participant is able to act upon the evaluation. Loss of the core positive or negative attitudes results in a dysfunctional compromise.

Holism, a coping mechanism, is action oriented and results when both the positive and negative attitudes can be accepted. It is likely the best approach to decreasing and resolving ambivalence. In the setting of ambivalence where the positive and negative attitudes are strong, wisdom, the result of prior experience, and learning moderates attitudinal differences. Wisdom is gained through mindfulness, both/and thinking and informed choice. Mindfulness occurs when a participant is open to new information, aware of multiple perspectives and can create new categories of meaning of positive and negative attitudes (37). Both/and thinking enables a participant to discover unconscious and conscious attitudes by actively comparing the positive and negative attitudes and discovering shared and distinct characteristics of them thereby undoing the suppression that occurs with the dominant response to ambivalence. Informed choice enables a participant to commit to an attitude (trust vs. mistrust), and the commitment results from knowledge of both the positive and negative attitudes that enable one to decide to trust or not. The positive effects of holism allow a participant to resolve ambivalence in a way that embraces both positive and negative attitudes, increases understanding of the attitude that results in a commitment, enables situational adaptability and positively influences the individual. There are several negative aspects of holism. It can decrease confidence and hinder action. It can increase ambivalence since both the positive and negative attitudes are kept. Finally, the final result of the holism response occurs over time during which the negative aspects of ambivalence can be more pronounced.

There are five factors that moderate ambivalence: free choice, information events, attitudes toward people compared to idea, safe relationships and role and task demands (14). Free choice determines whether ambivalence will result in commitment to trust or not to trust by increasing consistency and stability. Its influence lies in not forcing a participant to choose but, instead, allowing them to keep their ambivalence and reduce the negative effects of ambivalence over time. Information about a participant, relationship or situation that underlies ambivalence affects whether a participant will form positive or negative attitudes. Positive information results in a positive attitude while negative information produces a negative attitude. However, while the information itself is important, the quantity and intensity of the information whether positive or negative will directly influence whether one forms positive or negative attitudes. While attitudes toward people and ideas can both moderate ambivalence, those associated with people have more influence and will stimulate stronger desires to decrease ambivalence via less flexible responses. Safe family relationships or supportive social networks moderate ambivalence by providing strong support that can transform ambivalence about trusting to a commitment to trust. Finally, task and role demands moderate ambivalence in several ways. Integrative rather than distributive tasks favor the positive effects of ambivalence, are proactive in nature and create win-win situations for both parties. Role demands depend upon who has the power within a relationship and whether that participant is ambivalent. Ambivalent people with power cause those without power to disengage. Furthermore, the judgments that ambivalent empowered people make have little influence and are perceived to be of lower quality by the participant with lesser power. Recognizing and managing ambivalence is important and central to this process are the triggers that lead to ambivalence.

## Triggers of ambivalence

Understanding the triggers of ambivalence can yield an expedited path to ambivalence resolution. Sociologists and psychologists have varying views on the sources of ambivalence. Sociologists ascribe its origination to norms and roles, whereas psychologists attribute ambivalence to individual differences and relationships (34). Triggers of ambivalence can pertain to both individuals and organizations with each of them experiencing and operating in environments that are complex and dynamic. Since this research focuses on individuals and the ambivalence they experience, the triggers of ambivalence will be discussed from the individual perspective. Ashforth et al. (34) delineate four main types of triggers of ambivalence “(1)…contradictory goals and role conflicts; (2) membership dualities; (3) multifaceted objects; and (4) temporal factors” (34).

Competing expectations, beliefs, and behaviors can foster contradicting goals and conflicts in roles. Women in “high-status positions” can experience emotional ambivalence because from one aspect there is positive affect from attaining professional goals, and from another aspect negative affect can result from pressure to behave and conform to stereotypical gender roles (34). Contradictory goals manifest when the pursuit of one goal conflicts or undermines the pursuit of another goal, in other words, goal conflict (38).

Membership dualities provoke ambivalence because of the simultaneous requirement to do Activity A and to do the opposing action of Activity A (34). Dualities can be seen as oxymorons, paradoxes, ironies, and even dilemmas, hence triggering ambivalence (34, 39). A classic example of membership duality is the “paradox of belonging,” retaining individuality while adhering to group commonality. Another common duality is the “paradox of engaging,” trusting group members before trust is proven (34, 39).

Multifaceted objects evoke ambivalence because opposing attributes can surface over time. This attribute is often manifested in individuals' lives in the workplace and in relationships. The more knowledge attained and varied the experiences, the greater the storage of information and the greater the familiarity with the object, leading to encounters with the multiple imperfect facets. In other words, “Familiarity breeds ambivalence” (40).

Lastly, temporal factors give opportunities for “oppositional tendencies” which trigger ambivalence (34). Reflecting on past events conjures “counterfactual thinking” (34). A positive event can be reflected upon with an opposing statement such as “it could have been better” while a negative event can be characterized as, “it could have been worse” (34).

The explanation of these triggers elucidates the different social and psychological pathways to trigger ambivalence in individuals. Ambivalence causes the individual to be uncomfortable and stressed because of cognitive processing of the complex and dynamic attributes of the positive and negative elements and aspects of the object (27).

## Barriers to health care: external triggers of ambivalence

There are challenges to understanding ambivalence as it relates to health care, in general, and to clinical trial participation, in particular. First, in a patient-centered, health-care environment, it is necessary to understand the role that external factors may play as possible promoters and inhibitors in building and sustaining trusting relationships. And second, while there is a large body of research on trust, and a small body of research on distrust, our understanding of the role of ambivalence in the individual's clinical trial, decision-making process has yet to be explored. Critical to our understanding of how people *manage* ambivalence will be to understand how people use their experiences and interactions within the health-care system to navigate the process of *resolving* ambivalence.

The health care experience does not take place within a social vacuum. To fully understand, to measure, to improve and to enhance conditions of trust in clinical trial participation it is necessary to address the far-reaching implications of trust and distrust—as mediated by the construct of ambivalence—within a myriad of cultural, social, and interparticipantal relationships (10).

Prior research regarding the barriers to clinical trial participation indicates that individual health care experiences vary by race, ethnicity, gender, age, and geographic location, i.e., rural vs. urban residents (41). In current research, potential patients manage ambivalence by operationalizing barriers as trusting and distrusting relationships, i.e., as trust or distrust of the health-care system and/or ambivalence about the system, and even in terms of their relationship with the physician (42, 43).

There are many barriers to participation in clinical trials. Barriers such as the totality of previous health-care experiences, including lack of access to care, lack of transportation to receive care, prior experiences of health care discrimination, self-reported health status, income status, and level of education, (41, 44) can contribute to interpersonal ambivalence (20). Variables included in the following discussion that may have particular relevance in the management of ambivalence and clinical trial participation, also include the structure of the health-care system, and how health-related information is gathered, processed, and shared.

Interpersonal relationships “guided by past experiences and relationships that that may have been seen as trusting/distrusting and trustworthy/untrustworthy behavior” (10) may vary based on one's ethnicity, age or gender. It may only be possible to hypothesize about the impact of one's past experiences on one's level of trust (45, 46). Yet, past atrocities, such as slavery, infamous research trials, e.g., The U.S. Public Health Service Syphilis Study at Tuskegee, and the Guatemalan study continue to haunt the psyche of many Americans. For older individuals„ these atrocities seem to continue to inform current health choices (47). In Shavers et al. (47), 81 percent of African Americans, and 28 percent of white respondents “were aware” of the Tuskegee study. Forty-nine percent of the African Americans and 17 percent of whites indicated that this knowledge would negatively impact their decision to participate in a clinical trial (47). In studies of patient's reasons for non-participation in cancer clinical trials, lack of knowledge about cancer terminology, distrust of the medical system, and trust in physicians remained constant themes in focus group discussions about reasons for and against trial participation (42, 44). To manage ambivalence, Reich and Wheeler (14) found that in some instances people “cultivate ambivalence as an emotional hedge” in case what they desire may be out-of-reach. In short, if one has had negative experiences in the past or may have low expectations about their ability to be included or access a trial, ambivalence may be a useful emotion. Understanding the impact of the past, while developing new experiences that allow new stories of trust may be yet another method to begin to address ambivalence among prospective research participants.

Interpersonal relationships also may be mediated by the social context, which may be defined as the structure of the health-care system–to the extent that the system remains difficult to maneuver, difficult to access and quality of care may be dependent upon the patient's ability to pay or income status. (45, 48) It is imperative be mindful of the “the uniqueness of the health-care relationship,” (48) with the patient and the provider not sharing equal power in the relationship. Eliminating these disparities is politically sensitive and challenging (49). The health-care system—as a whole—may be viewed in society as a continuation of structures and policies that produce inequalities in general (49). One primary example is the inconsistency of current health-care legislation. In one instance, health policy sought to increase the inclusion of women and the underserved in accord with the 1993 Legislation, which called for an increase in clinical trial participation. Legislation such as the Affordable Care Act, which includes a provision to allow private insurers to cover the cost of some routine patient care for participation in cancer clinical trials, has resulted in claim denials, which may prove to be yet another barrier to clinical trial participation (50). Ambivalence may be triggered by uncertainty. Uncertainty is a response to lack of information and knowledge, which may make it difficult to know how to feel about an issue (14).

Changes in information gathering as a moderator of interpersonal ambivalence—in an age of burgeoning social media usage and reliance—has enormous implications for gathering, assessing, and accepting health information. To date, few studies have explored the impact of social media usage for information seeking as enhancing or diminishing interpersonal ambivalence (32). How information about a vast number of experiences—to which we may not be privy, but to which we now may feel connected—is shared and processed may take on greater meaning in the future “when constructing informational environments” (32). Ambivalent opinions may be tempered by several variables, including attitude strength, which information to seek or ignore, cognitive dissonance, and the amount of participant knowledge about an issue (32). How the use of social media, e.g., Facebook, Instagram, WhatsApp, Twitter, has negatively or positively engendered public trust gives rise to numerous questions (32). Mixed messages about health confound the public trust (51). What impact did social media have—in retrospect—in shaping the saliency and information-seeking activities among various groups about issues such as vaccine, or research trial safety? McConnel (52) in his review of the role of mental health treatment and social support, concludes that “individuals draw social support from their relationships.” Is electronic connectivity a fulfilling relationship in terms of what we now define as social support? People exercise the ability to seek information based on individual attitudes and motivation (32). Among the various cultures, what impact will social media have on information seeking behavior? African Americans' beliefs about white society's devaluation of black lives and ongoing conspiracy against the race has had a long-lasting impact on relationships with the health profession (53). In the era of “fake news,” how does one determine what is real and what is not?

Access to different electronic channels, provides the opportunity to strengthen communication and the capacity of providers to serve the underserved (49). Ambivalence among individuals/patients does not remain static. The individual/patient can change rapidly both positively and negatively and reverse again slowly or just as rapidly. This rate and speed of change is where social media can act as a retardant or accelerant in shaping positive or negative opinions. In light of the above considerations, managing and mediating patient ambivalence in health care settings is an important component of clinical trial participation.

## Motivational interviewing

In this review, trust, distrust, and ambivalence have been clearly delineated in relation to clinical trials participation. Furthermore, individual triggers and societal factors affecting ambivalence have also been examined. The relationship between these factors ultimately impacts voluntary participation in clinical trials. Voluntary patient participation, based on the premise of a patient's trust, is a crucial aspect of clinical trial participation and healthcare treatment. Ambivalence is a normal and uncomfortable component of the complex decision-making process when one is considering a change (54, 55). It is necessary, then, to create opportunities for change by reducing ambivalence. Increasing trust includes assisting patients to ameliorate ambivalence around shared-decision making with their clinician, as well as make changes in participant behavior (17). Changing behaviors and making decisions arises from addressing and managing a patient's level of ambivalence. This includes (1) *engaging;* creating a relationship between the clinician and patient; (2) *focusing*; exploring the direction of change; (3) *evoking;* eliciting the patient to state their own reasons for changing; and (4) *planning;* creating an action plan (17). By addressing a patient's own reasons for ambivalence toward making a decision or preparedness to make a change, a provider can use “change talk” to reduce that patient's resistance to participating in treatment (56). Motivational Interviewing (MI) focuses on using therapeutic techniques to address such ambivalence in relation to change and has widespread applications in healthcare settings.

MI allows the clinician to engage with a patient collaboratively, rather than using a prescriptive or action-oriented approach. Research reports the use of five specific skill-based strategies used within the MI framework to address ambivalence. These include open-ended questions, affirmations, reflections, and summaries, also known as “OARS” (56). MI employs active listening skills, including reflecting what the participant has said and asking questions without specific “yes” or “no” answers (57). Rather than asking a patient to address an issue with a specific answer, such as a closed question, MI allows the patient to answer a question on his or her own terms and to self-identify the reasoning behind his or her willingness to change a certain action or behavior in relation to his or her healthcare treatment (56). By demonstrating to the patient that the clinician has compassion, empathy, and a clear understanding of that patient's multi-faceted problem, which may involve many levels of inconsistencies that promote feelings of ambivalence (23), motivational interviewing allows the patient to fundamentally address his or her readiness for change (58).

MI can address a wide variety of behaviors related to treating chronic health diseases (16). More specifically, there is evidence that may suggest MI can assist patients with cancer to address psychosocial needs, as well as contemplate changes in lifestyle behaviors, such as diet, exercise, and smoking cessation (58). In a meta-study that evaluated 48 studies utilizing MI as an intervention tool, the use of MI increases the chance of a positive outcome by 55%. Positive outcomes include changes in physical prognostic markers, decreased alcohol and substance use, and increases in following medical advice, such as monitoring blood sugar levels, increasing physical activity, and increased confidence on managing chronic health conditions. However, there were other outcomes that had mixed results or that had negative results based on MI, including behaviors that focus on risk-reduction, other physical functioning outcomes, and markers of quality of life, such as depression, anxiety, pain, and adjustment to diseases (59).

Research has even shown that MI techniques have been used by practice oncology nurses for pain management in patients with cancer (60). In addition, MI may benefit cancer survivors, including helping patients to lose weight, increase overall quality of life, and improve clinical biomarkers (61). Furthermore, when MI is provided for patients undergoing treatment for cancer, there may be a higher likelihood that patients will feel optimistic about the future and increase confidence around participant healthcare management and decision-making (62). MI may also assist with increasing health screenings outcomes for patients who could potentially have cancer, among other diseases and illnesses (63). Additionally, MI has applications in clinical trials and has been utilized in a various studies, including substance abuse, homelessness, and medication adherence. For instance, MI has been effective in clinical trials at promoting smoking cessation (64). Motivational interviewing has even been shown to promote changes in behavior that lead to increased medication adherence (65). Clearly, there is a significant amount of empirically-supported evidence suggesting that clinicians who utilize MI in practice are actively decreasing ambivalence, establishing strong rapport and trust with their patients, as well as increasing patients' overall quality of life. Ultimately, this enables these patients to make healthcare decisions that are based on self-efficacy and self-determination. However, in the healthcare industry, there may be little awareness about how to utilize motivational interviewing among front-line professionals (66).

There is also little information in the literature about the relationship between MI and trust. While there is supporting literature that conceptualizes the relationship between trust and ambivalence, as has been discussed in this article, there is little evidence that directly evaluates the impact of MI on levels of trust. Given the evidence on the role of MI in managing ambivalence in a multitude of studies and clinical trials, it is plausible to consider that MI also has an impact that would lead to increased trust and, presumably, decreased distrust. Future studies should consider the role of motivational interviewing and its relationship to trust, distrust, and ambivalence. Such studies should also evaluate the use of MI in clinical oncology trials, as well as other cancer-related treatment and prevention programs. Such studies should emphasize the role of MI in reducing ambivalence as well as increasing trust, engagement, and rapport between the client and patient. There are also a myriad of other outcomes that could be considered for such studies, including increases or improvements in participation rates for vulnerable and marginalized populations, perceived quality of treatment, as well as participants' willingness to change lifestyle factors impacting healthcare management.

## Author contributions

All authors (JC, MR, JL, LJ, CK, and TH) contributed to the conception and design of the study. JC, JL, MR, and TH shared equally in writing the manuscript. All authors contributed to manuscript revision, read, and approved the submitted version.

### Conflict of interest statement

The authors declare that the research was conducted in the absence of any commercial or financial relationships that could be construed as a potential conflict of interest.
